# Supplementary material for: Treatment Outcomes of Adolescents Compared to Younger Pediatric Patients with Acute Myeloid Leukemia: Do They Need a Special Approach?
Source: Cancers (Basel). 2024 Mar 14;16(6):1145. doi: 10.3390/cancers16061145 (PMC10969545; doi:10.3390/cancers16061145)
Supplement: Supplementary file 1 [file cancers-16-01145-s001.zip › cancers-2884623-supplementary.pdf]

**Figure S1. The details of therapeutic regimens according to AML-BFM-2012 Registry and AML-BFM-2019 protocols**

**AML-BFM 2012 Registry**

| | | | | | | |
|---|---|---|---|---|---|---|
| **SRG inv(16)** | AIE | AI | hAM | HAE | Maintenance | |
| **SRG** | AIE | HAM | AI | hAM | HAE | Maintenance |
| **IRG** | AIE | HAM | AI/2-CDA | hAM | HAE | Maintenance |
| **HRG** | AIE | HAM | AI/2-CDA | hAM | alloHSCT | |

**AML-BFM 2019**

| | | | | | | |
|---|---|---|---|---|---|---|
| **SRG inv(16)** | AIE | AI | hAM | HAE | Maintenance | |
| **SRG** | AIE | HAM | AI | hAM | HAE | Maintenance |
| **IRG** | AIE | HAM | AI | hAM | HAE | Maintenance |
| **HRG** | AIE | HAM | AI | hAM | alloHSCT | |

**Induction phases:**
**AIE:** cytarabine 100 mg/m2/day continuous infusion on days 1 and 2, followed by 30 min infusion every 12 h on days 3–8; idarubicin 12 mg/m2/day, 30 min infusion days 3, 4, and 5; etoposide 150 mg/m2/day, 60 min infusion on days 6–8.
**HAM:** cytarabine 3 g/m2/dose, 3 h infusion every 12 h on days 1–3 (6 doses); mitoxantrone 10 mg/m2/day, 30 min infusion on days 3 and 4.

**Consolidation phases:**
**AI/2-CDA:** cytarabine 500 mg/m2/day, 96-h infusion on days 1–4; idarubicin 12 mg/m2/day, 30 min infusion on days 3 and 5; 2-chloro-2-deoxyadenosine 6 mg/m2/day, 30 min infusion, on days 1 and 3.
**AI:** cytarabine 500 mg/m2/day, 96-hours infusion on days 1–4; idarubicin 12 mg/m2/day, 30 min infusion on days 3 and 5.
**hAM:** cytarabine 1 g/m2/dose, 3 h infusion every 12 h on days 1–3 (6 doses); mitoxantrone 10 mg/m2/day, 30 min infusion on days 3 and 4.
**HAE:** cytarabine 3 g/m2/dose, 3 h infusion every 12 h on days 1–3 (6 doses); etoposide 125 mg/m2/day, 60 min infusion on days 2–5.

**Maintenance:**
AML-BFM 2012: daily thioguanine 40 mg/m2/day, orally; cytarabine 40 mg/m2/day, i.v. or s.c., 4 days every 4 weeks for 1 year.

**Table S1.** Stratification to risk group according to treatment protocols.

| Protocol | AML-BFM 2012 Registry | AML-BFM 2019 |
|---|---|---|
| **Standard risk group (SRG)** | t(8;21), inv(16), t(1;11), NPM1, CEBPdm[a] | Inv(16)(p13.1q22)<br>t(16;16)(p13;q22)<br>t(8;21)(q22;q22)<br>t(1;11) (q21;q23)<br>Normal karyotype and NPM1-mutation<br>Normal karyotype and CEBPA (double mutation)[a] |
| **Intermediate risk group (IRG)** | All others [b] | All others[b] |
| **High risk group (HRG)** | t(4;11), t(5;11), t(6;11), t(10;11), t(6;9), t(7;12), der12p, isolated monosomy 7, t(9;22), FLT3-ITD and WT1mut, complex karyotype | 12p/ t(2;12), 5/5q,<br>FLT-ITD and WT1mut,<br>7 (not in combination with favorable/MLL-aberrations),<br>t(4;11)(q21;q23); KMT2A::AF4,<br>t(5;11)(q35.3;p15);NUP98::NSD1,<br>t(6;11)(q27;q23);KMT2A::AF6,<br>t(10;11)(p12;q23);KMT2A::AF10,<br>t(6;9)(p23;q34), t(7;12)(q36;p13),<br>t(9;22)(q34;q11), complex karyotype,<br>inv(3)(q21q26.2)/t(3;3)(q21;q26.2),<br>t(16;21)(p11;q22);FUS::ERG,<br>Inv(16)(p13.3q24.3);CBFA2T3::GLIS2 |

[a] In case of bone marrow blasts >20% on the days 21-28—reclassification to IRG
[b] In case of bone marrow blasts > 20% on the days 21-28 or >5% on the days 42-56—reclassification to HRG
